# Control Work

**Published:** 1898-10

**Authors:** 


### CONTROL WORK.

*Kentucky.*—When the food contains poisonous ingredients, or when it contains antiseptics or preservatives not known to the purchaser; when it consists in whole or in part of diseased or putrid substances, or is in any part the product of a diseased animal, or an animal that has died otherwise than by slaughter, it is not to be offered for sale. The law went into effect June 30th, last.—*Abstract from Pure Food Law.*

*California.*—Inspectors of the Bureau of Animal Industry are authorized to grant special permits, in accordance with the instructions of the chief of that bureau, for the movement of cattle from the State to other States after the cattle have been inspected and found free from infection. This is on account of the severity of the drought in southern parts of California, where they are slaughtering cattle because of lack of feed and water.

Special order of the Bureau of Animal Industry: No vessels shall be permitted to take on board any cattle or sheep unless the same have been allowed at least twelve hours' actual rest in the yards at the port of embarkation before the vessel sails, nor until the loading of the other cargo has been completed.

